# The protective effect of PFTα on alcohol-induced osteonecrosis of the femoral head

**DOI:** 10.18632/oncotarget.19160

**Published:** 2017-07-11

**Authors:** Yi-Xuan Chen, Dao-Yu Zhu, Jun-Hui Yin, Wen-Jing Yin, Yue-Lei Zhang, Hao Ding, Xiao-Wei Yu, Jiong Mei, You-Shui Gao, Chang-Qing Zhang

**Affiliations:** ^1^ Department of Orthopedic Surgery, Shanghai Jiao Tong University Affiliated Sixth People’s Hospital, Shanghai 200233, China; ^2^ Institute of Microsurgery on Extremities, Shanghai 200233, China; ^3^ Department of Orthopedic Surgery, The Second Affiliated Hospital of Wenzhou Medical University, Wenzhou, Zhejiang 325027, China

**Keywords:** ethanol, Wnt/β-catenin pathway, osteonecrosis of the femoral head, BMSC, PFTα

## Abstract

Epidemiologic studies have shown alcohol plays a pivotal role in the development of osteonecrosis of the femoral head (ONFH). The aim of this study was to explore the underlying mechanism of alcohol-induced ONFH and the protective effect of pifithrin-α (PFTα). In vitro, we found ethanol treatment significantly activated p53, suppressed Wnt/β-catenin signaling and inhibited osteogenic-related proteins. Furthermore, by separating the cytoplasmic and nuclear proteins, we found ethanol inhibited osteogenesis by impairing the accumulation of β-catenin in both the cytoplasm and nucleus in human bone mesenchymal stem cells (hBMSCs), which resulted from activating glycogen synthase kinase-3β (GSK-3β). Therefore, PFTα, a p53 inhibitor, was introduced in this study to block the ethanol-triggered activation of p53 in hBMSCs and alcohol-induced ONFH in a rat model. In vivo, we established alcohol-induced ONFH in rats and investigated the protective effect of PFTα. Hematoxylin & eosin (H&E) staining combined with TdT-mediated dUTP nick end labeling (TUNEL), cleaved caspase-3 immunohistochemical staining, and micro-CT images revealed substantial ONFH in the alcohol-administered rats, whereas significantly less osteonecrosis developed in the rats injected with PFTα. Osteogenic-related proteins, including osteocalcin, osteopontin and collagen I, were significantly decreased in the alcohol-administered rats, whereas these results were reversed in the PFTα-injected rats. Fluorochrome labeling similarly showed that alcohol significantly reduced the osteogenic activity in the rat femoral head, which was blocked by the injection of PFTα. In conclusion, PFTα had an antagonistic effect against the effects of ethanol on hBMSCs and could be a clinical strategy to prevent the development of alcohol-induced ONFH.

## INTRODUCTION

Alcohol consumption is recognized as one of the leading risk factors of atraumatic osteonecrosis of the femoral head (ONFH). Ultimately, ONFH will cause structural collapse, leading to hip joint dysfunction. Patients who develop ONFH will eventually undergo total hip arthroplasty. Various mechanisms, including osteogenic impairment, oxidative stress, fat embolism, and hyperlipidemia, have been related to the pathology of alcohol-induced bone metabolism [1–7]; however, the exact pathogenic process by which alcohol induces ONFH development has not yet been clarified.

Several recent studies identified impaired bone synthesis and decreased bone mineral density (BMD) in patients with alcohol-induced ONFH [2, 8–12]. Similar observations of decreased osteogenic responses were detected in alcohol-administered animals [3, 7, 13]. In an *in vitro* study, ethanol was reported to significantly impair the osteogenic differentiation of bone mesenchymal stem cells (BMSCs), which play a pivotal role in bone regeneration and repair [14]. Hence, the decreased osteogenesis of BMSCs might be an underlying mechanism of alcohol-induced ONFH.

P53 is known to play a pivotal role in cell processes such as genomic stability, growth, proliferation and immunity [15–17]. Previous studies have demonstrated that p53 possesses inhibitory effects on osteogenesis, bone mesenchymal stem cells and bone remodeling both *in vivo* and *in vitro* [18–20]. Moreover, knockout of the p53 gene in the mouse model resulted in enhanced osteogenesis [21]. A recent study demonstrated that ethanol could induce the activation of p53 *in vitro* [22]. Therefore, we consider that the p53 gene might be a critical mediator of ethanol-triggered anti-osteogenesis and may play a role in the development of alcohol-induced ONFH. Hence, an inhibitor of p53 may have a protective role in the anti-osteogenic effect following alcohol administration and thereby prevent ONFH development. PFTα is a small molecule that inhibits the accumulation of p53 by decreasing its stability and reducing p53-associated gene transcription [23]. PFTα was shown to protect neurons against traumatic brain damage [24], enhance the resistance of hepatocytes to genotoxic agents [25], protect renal tubular epithelial cells against injury [26], and alleviate apoptosis and acute cardiotoxicity in mice [27]. However, the effect of PFTα on alcohol-induced ONFH is not clear.

In this study, a series of *in vitro* assays were employed to reveal the activation of p53, the de-activation of Wnt/β-catenin and the impairment of osteogenesis by ethanol treatment in hBMSCs. Co-administration of PFTα abolished the stimulatory effect of ethanol on p53 and restored the osteogenic differentiation potential of BMSCs. Additionally, we found that this inhibitory effect of p53 on osteogenesis was associated with the phosphorylation of GSK-3β, which was rescued by PFTα. Using multiple assessments, we observed significantly increased osteogenesis and a lower incidence of alcohol-induced ONFH in animals co-treated with PFTα. Hence, we demonstrated that the ethanol-triggered upregulation of p53 activity was associated with the suppression of osteogenic differentiation via the Wnt/β-catenin pathway, which contributed to the development of alcohol-induced ONFH; moreover, the p53 inhibitor PFTα may be considered a rescue strategy.

## RESULTS

### PFTα treatment rescued the inhibitory effect of ethanol on the osteogenic differentiation of hBMSCs

**Figure 1:**
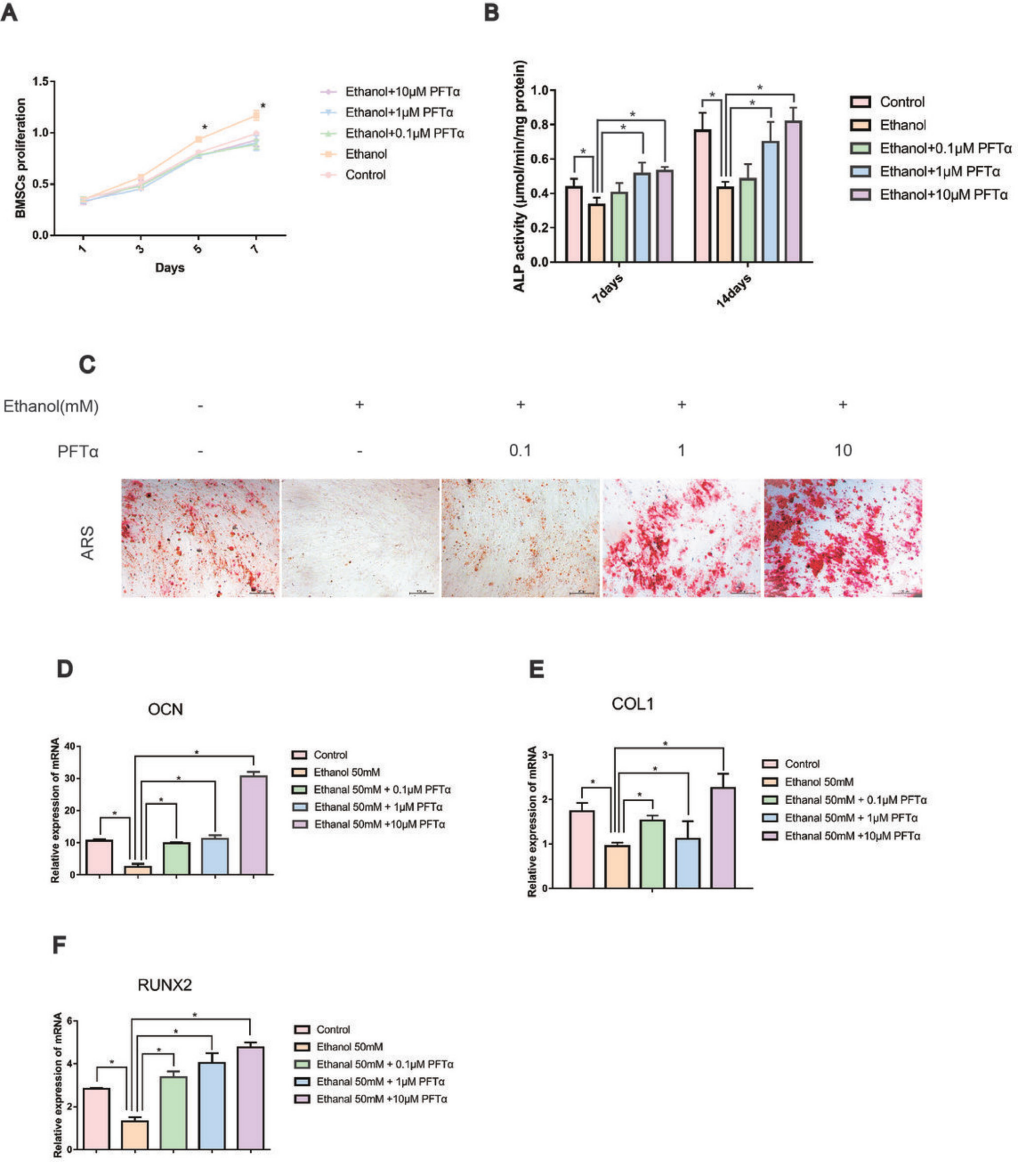
PFTα treatment rescued the inhibitory effect of ethanol on the osteogenic differentiation of hBMSCs (**A**) hBMSCs were incubated for 1, 3, 5 and 7 days in a medium supplemented with 50mM and/or different concentrations of PFTα as indicated, and the proliferation of hBMSCs was recorded. Values are shown as the means ± SD (*N* = 3). (**B**) The ALP activity of hBMSCs was recorded after the hBMSCs were cultured for 7 and 14 days in an osteogenic medium supplemented with 50mM ethanol and/or different concentrations of PFTα as indicated. Values shown are the means ± SD (*N* = 3 for each group; ^*^significant difference versus the 50-mM ethanol group). (**C**) Alizarin red staining (ARS) was performed after a 14-day incubation period in osteogenic media. Whereas 50 mM of ethanol profoundly reduced the calcium deposits, higher concentrations of PFTα rescued the ethanol-induced anti-osteogenic effects. (**D–F**) The relative osteogenic-related gene expressions normalized to β-actin were decreased by 50 mM of ethanol after 24 hours and rescued by different concentrations of PFTα as indicated. (*N* = 3 for each group; ^*^significant difference versus the 50-mM ethanol group).

BMSCs are the major source of bone formation and regeneration [28]. First, ethanol treatment slightly improved the proliferation of hBMSCs compared to that in the other groups, as shown in Figure 1A, with no significant difference among the other groups. To evaluate the effects of ethanol on the osteogenic responses of BMSCs *in vitro*, ARS was performed and the alkaline phosphatase (ALP) activity was determined. As previously suggested, an ethanol concentration of 50 mM was used in our experiment [4]. The ALP activity of hBMSCs was significantly impaired by ethanol treatment (Figure 1B, ^*^p < 0.05) but reversed by PFTα (^*^p < 0.05). The calcium mineral deposition in hBMSCs treated with ethanol (50 mM) was profoundly reduced compared with that in the control group. With increased PFTα concentration, the anti-osteogenic effects of ethanol were abolished (Figure 1C).To verify the effect of PFTα on the osteogenic differentiation of hBMSCs, we identified osteogenic-associated gene expression, including RUNX2, OCN, and COL1 using RT-PCR. We found that the expression levels of RUNX2, OCN and COL1 in the ethanol group were lower than those in control group, while different concentrations of PFTα from 0.1 μM to 10 μM significantly reversed the inhibitory effects (Figure 1D–1F). Among the PFTα-treated groups, RUNX2, OCN and COL1 were most significantly upregulated by 10 μM PFTα (Figures 1D–1F).

**Figure 2:**
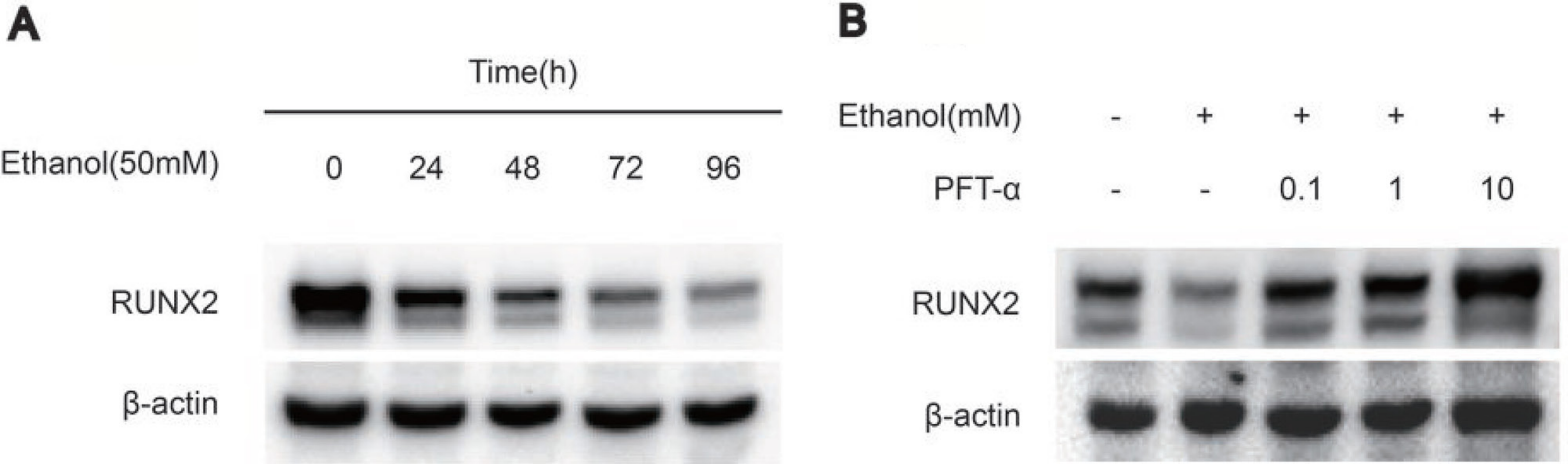
PFTα treatment rescued the inhibitory effect of ethanol on osteogenic-related protein expression in hBMSCs (**A**) The Western blot analysis of RUNX2 expression indicated that 50mM ethanol decreased the osteogenic differentiation of hBMSCs in a time-dependent manner. Osteogenic-induced hBMSCs were treated with 50mM ethanol for the time indicated. (**B**) The Western blot analysis of RUNX2 indicated that PFTα reversed the anti-osteogenic effect of ethanol in hBMSCs. Osteogenic-induced hBMSCs were cultured for 72 hours in a medium supplemented with 50mM ethanol and/or different concentrations of PFTα as indicated. β-actin was used as an internal control.

**Figure 3:**
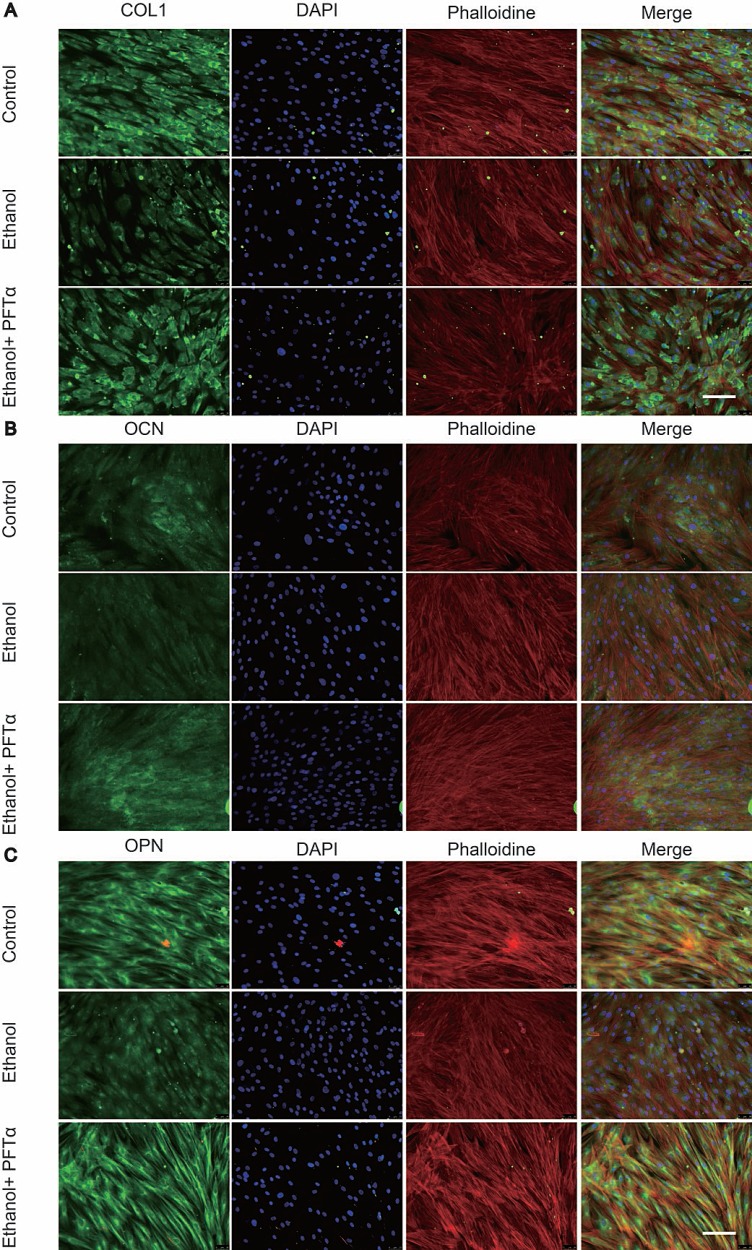
PFTα treatment rescued the inhibitory effect of ethanol on osteogenic-related protein expression in hBMSCs (**A**) Immunofluorescence staining of COL1 showed PFTα reversed the anti-osteogenic effect of ethanol in hBMSCs. (**B**) PFTα increased positive staining of OCN. (**C**) Immunofluorescence staining of OPN was increased by PFTα. hBMSCs were cultured for 72 hours in an osteogenic differentiation medium supplemented with 50 mM ethanol and/or 10 μM PFTα and immune-stained with COL1, OCN and OPN. Cytoskeletons were stained with phalloidine (red), and the nucleus was stained with DAPI (blue).

To explore the effect of ethanol on the expression of osteogenic proteins in hBMSCs, we performed Western blotting of RUNX2. The results showed that ethanol treatment significantly decreased RUNX2 protein levels in hBMSCs in a time-dependent manner (Figure 2A). Additionally, the level of RUNX2 was restored by different concentrations of PFTα (Figure 2B). A higher concentration (10 μM) of PFTα reversed the anti-osteogenic effect of ethanol most notably, which was in accordance with our RT-PCR results. The downstream targets of RUNX2 [29], including OCN, OPN and COL1, were detected in hBMSCs by immunofluorescence staining. The hBMSCs were treated with 50 mM ethanol and/or 10 μM PFTα for 72 hours and immunostained with COL1, OPN and OCN. The results showed that ethanol treatment significantly decreased COL1, OPN and OCN staining, whereas increases in those osteogenic-related proteins were observed with PFTα supplementation (Figure 3A–3C).

### The rescue effect of the osteogenic potential of PFTα on hBMSCs was associated with the p53/Wnt/β-catenin pathway

**Figure 4:**
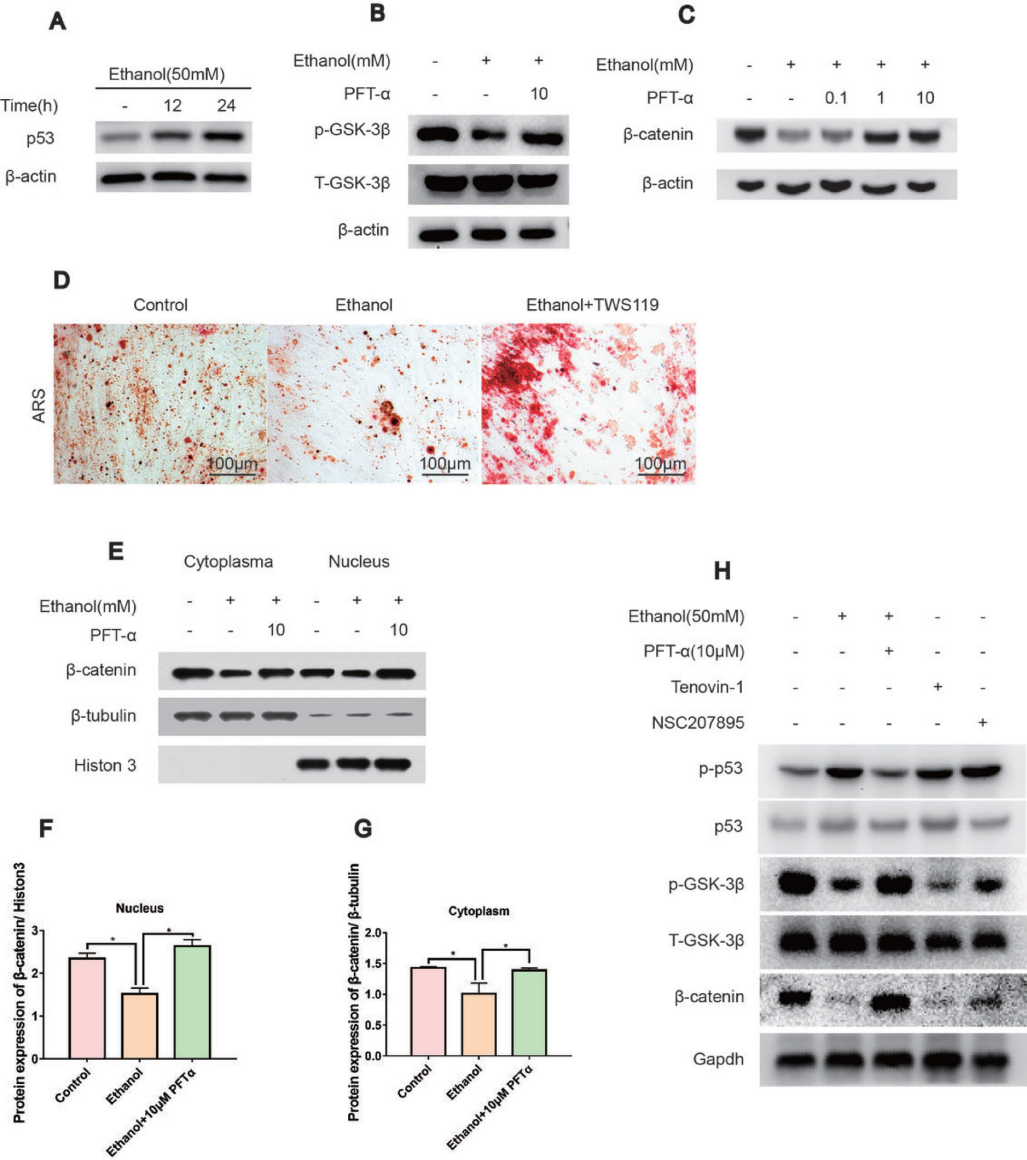
The rescue effect of the osteogenic potential of PFTα on hBMSCs was associated with the Wnt/β-catenin pathway (**A**) The p53 protein expression was increased by ethanol in a time-dependent manner. hBMSCs were treated with 50 mM ethanol and/or 10 μM PFTα for the time indicated. (**B**) PFTα reversed the inhibitory effect of ethanol on GSK-3β phosphorylation. Cells were treated with 50 mM ethanol and/or 10 μM of PFTα for 24 hours and Western blotted for p-GSK-3β and total-GSK-3β. Total-GSK-3β was used as an internal control. (**C**) The total amount of β-catenin decreased by ethanol was rescued by PFTα in hBMSCs. Cells were treated for 24 hours with 50 mM ethanol and/or different concentrations of PFTα as indicated and Western blotted for β-catenin. β-actin was used as an internal reference. (**D**) BMSCs were cultured in osteogenic-inducing medium supplemented with ethanol (50 mM) and/or GSK3β inhibitor TWS119 (10 μM) for 21 days and stained for calcium deposits. The ethanol induced anti-osteogenic effect on BMSCs was restored by GSK3β inhibition. (**E**) The separation of proteins in the nucleus and cytoplasm of hBMSCs showed decreased nuclear translocation of β-catenin by ethanol, which was reversed by 10 μM of PFTα. hBMSCs were treated with 50mM ethanol and/or 10 μM of PFTα for 24 hours and Western blotted for β-catenin in the cytoplasm and nucleus. β-tubulin was used as an internal reference for cytoplasm proteins, and Histon-3 was used as the normalization control for nuclear protein. (**F–G**) The quantification of β-catenin expression in the cytoplasm and nucleus was performed using ImageJ software (^*^significant difference versus the 50-mM ethanol group). (**H**) hBMSCs were treated with ethanol (50 mM), PFTα (10 μM), Tenovin-1(10 μM) and NSC207895(10 μM) for 24 hours and immune-blotted for indicated protein. Gapdh served as an internal reference.

Using immunoblotting, we found that the protein level of p53 was increased by ethanol treatment in a time-dependent manner (Figure 4A). To probe the regulation of ethanol and PFTα on Wnt/β-catenin, we assessed GSK-3β activity, which is the main mediator of the β-catenin degradation pathway. We found that the GSK-3β phosphorylation level was decreased after ethanol treatment and restored by PFTα (Figure 4B). As the downstream target, the β-catenin protein level was decreased by ethanol and was rescued by different concentrations of PFTα in the hBMSCs (Figure 4C). A higher concentration (10 μM) of PFTα increased the total β-catenin level most significantly (Figure 4C). We also performed Alizarin Red staining of BMSCs to investigate the role of GSK-3β in the osteogenic differentiation of BMSCs. Our results indicated that a selective GSK-3β inhibitor, TWS119 (10 μM), alleviated the ethanol-induced anti-osteogenic effect (Figure 4D). Furthermore, the separation of proteins in the nucleus and cytoplasm of hBMSCs was performed. The results showed a decrease in nuclear β-catenin levels by ethanol treatment, and supplementation with 10 μM of PFTα restored the nuclear localization of β-catenin (Figure 4E). Quantification of β-catenin expression was performed using ImageJ software (Figures 4F-4G). Herein, our results suggested that ethanol impaired the translocation of β-catenin from the cytoplasm to the nucleus via the activation of GSK-3β, which was reversed by PFTα.

To mimic the effect of ethanol on p53/Wnt/β-catenin signaling, selective p53 activators, including Tenovin-1 and NSC207895, were introduced [30, 31]. We found that both Tenovin-1 and NSC207895 upregulated p-p53 and p53, which indicated activation of p53. In contrast, downregulation of p-GSK-3β and β-catenin in hBMSCs suggested that the rescue effect by PFTα on hBMSCs was associated with p53/Wnt/β-catenin signaling (Figure 4H).

### The development of alcohol-induced ONFH in the rat model

The rat ONFH model was established by feeding the animals a Lieber-Decarli liquid diet, as described in previous studies [32, 33]. Signs of femoral head osteonecrosis were assessed by histologic examinations. Histologic evidence of ONFH was based on H&E staining, TUNEL staining and cleaved caspase-3 immunohistochemical staining of paraffin-embedded sections. We defined osteonecrosis as diffuse empty lacunae or the presence of pyknotic nuclei in bone trabeculae accompanied by surrounding bone marrow cell necrosis [34–36].

**Figure 5:**
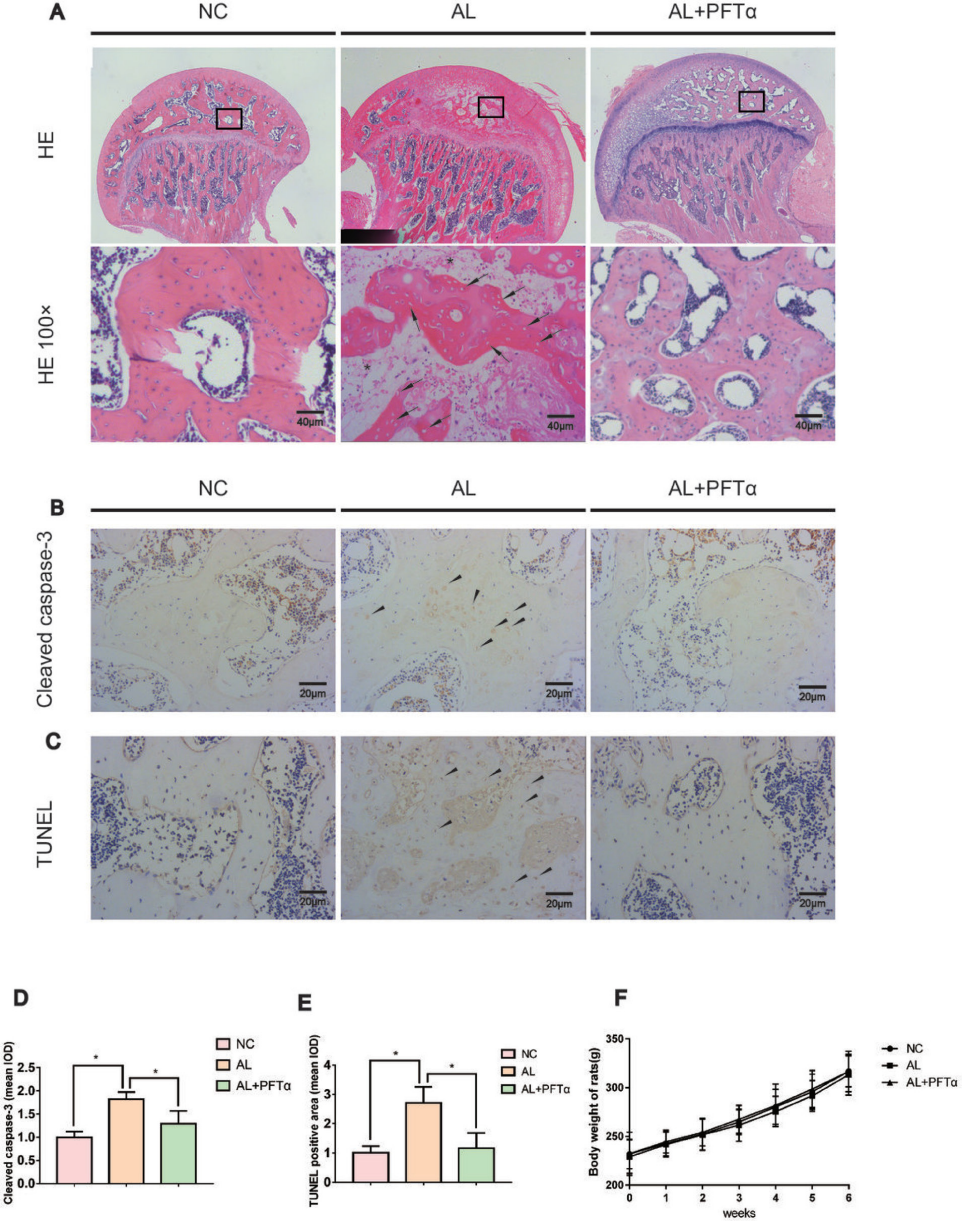
The development of alcohol-induced ONFH in the rat model (**A**) H&E staining of the femoral head indicated obvious signs of osteonecrosis in the AL group. Empty lacunae or pyknotic nuclei (black arrows) were present in the subchondral trabeculae of the AL group, and the accumulation of bone marrow hematopoietic cellular debris was present in the medullary space (black stars). Significantly less ONFH change was detected in the AL+PFTα group. (**B**) Immunostaining of cleaved caspase-3 in the femoral heads is shown. Positive staining was present in the trabeculae of the femoral head in the AL group (black triangles). (**C**) TUNEL staining of the femoral head is shown. Positive staining was present in the trabeculae of the femoral head in the AL group (black triangles). (**D–E**) Quantification of positive staining of cleaved caspase-3 and TUNEL. (*N* = 3, ^*^significant difference versus the AL group, ^*^p < 0.05) (**F**) No significant difference in body weight was observed among the three rat groups.

H&E staining showed diffuse empty lacunae and pyknotic nuclei in bone trabeculae with the accumulation of bone marrow hematopoietic cellular debris in the medullary space in the AL group after six weeks of alcohol administration (Figure 5A). With PFTα treatment however, significantly fewer osteonecrotic lesions were observed (Figure 5A). No apparent ONFH histopathologic change was found in the NC group.

Apoptosis is a crucial pathological change in ONFH development [37–39]. Herein, we performed cleaved caspase-3 immunostaining in the rat femoral head. Significant positive staining was observed in the trabecular bone of the AL group and was attenuated in the AL+PFTα group (Figure 5B, 5D). Similar results were observed with TUNEL staining (Figure 5C, 5E), which suggested signs of apoptosis in the femoral heads of the alcohol-administered rats. No significant difference in body weight was observed among the three rat groups (Figure 5F).

### The decreased alcohol-induced osteogenic activity was rescued by PFTα *in vivo*

**Figure 6:**
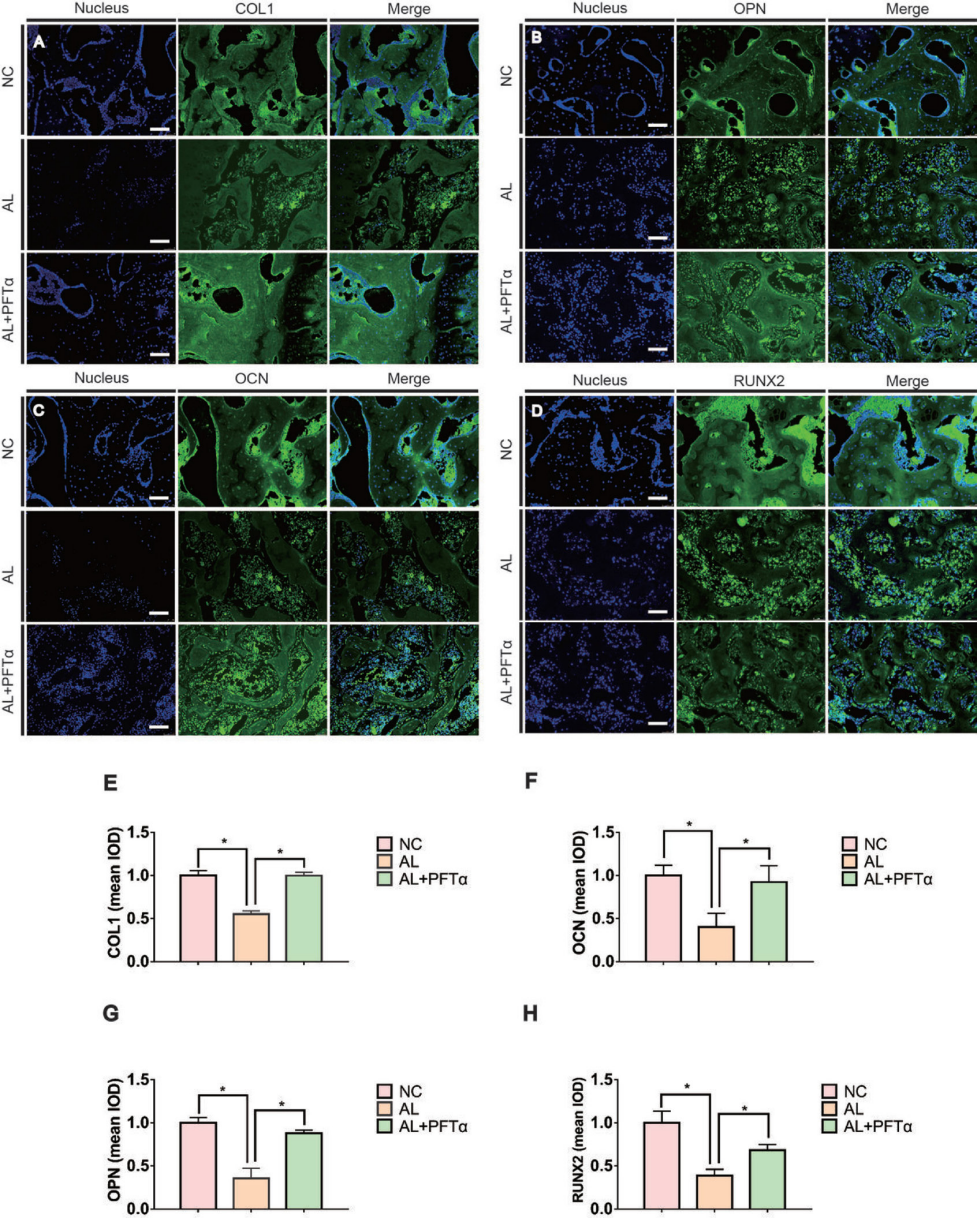
Alcohol-induced decreased osteogenic activity was rescued by PFTα *in vivo* (**A–D**) Immunohistochemical staining for OPN, COL1, RUNX2 and OCN in coronal sections of the femoral heads showed significantly reduced target protein expression in the subchondral trabeculae in the AL group. PFTα-injected rats showed a significant increase in positive staining compared to that in the AL group. The white triangles indicated the target protein staining. The yellow triangles indicated the empty lacunae. (**E, F, G, H**) Positive staining of OPN, COL1, RUNX2 and OCN in the femoral heads was quantified using Image-Pro Plus. (*N* = 3, ^*^significant difference versus the AL group, ^*^p < 0.05).

To assess the effect of alcohol administration and PFTα injection on bone-related protein expression, immunofluorescence staining of OPN, OCN, RUNX2 and COL1 was performed. Proteins such as OPN, OCN, RUNX2 and COL1 are known to be osteogenesis-related markers expressed during osteogenic differentiation and calcium mineralization [40]. We found a significant decrease in OPN-, OCN-, RUNX2- and COL1-positive staining in the femoral heads of the AL group compared to the NC group (Figures 6A–6D), whereas the AL+PFTα group showed a significant increase in protein expression compared to that in the AL group (Figures 6A–6D). After immunofluorescence staining of these proteins, we analyzed the results using Image-Pro Plus software. Figures 6E-6H show the calculated positive staining of the proteins indicated. Notably, we also observed diffuse empty lacunae and significantly fewer cell nuclei in the trabecular bone in the AL group than in that in the NC group. This finding was in accordance with our H&E results.

### Micro-CT analyses showed alleviation of alcohol-induced ONFH with PFTα treatment

**Figure 7:**
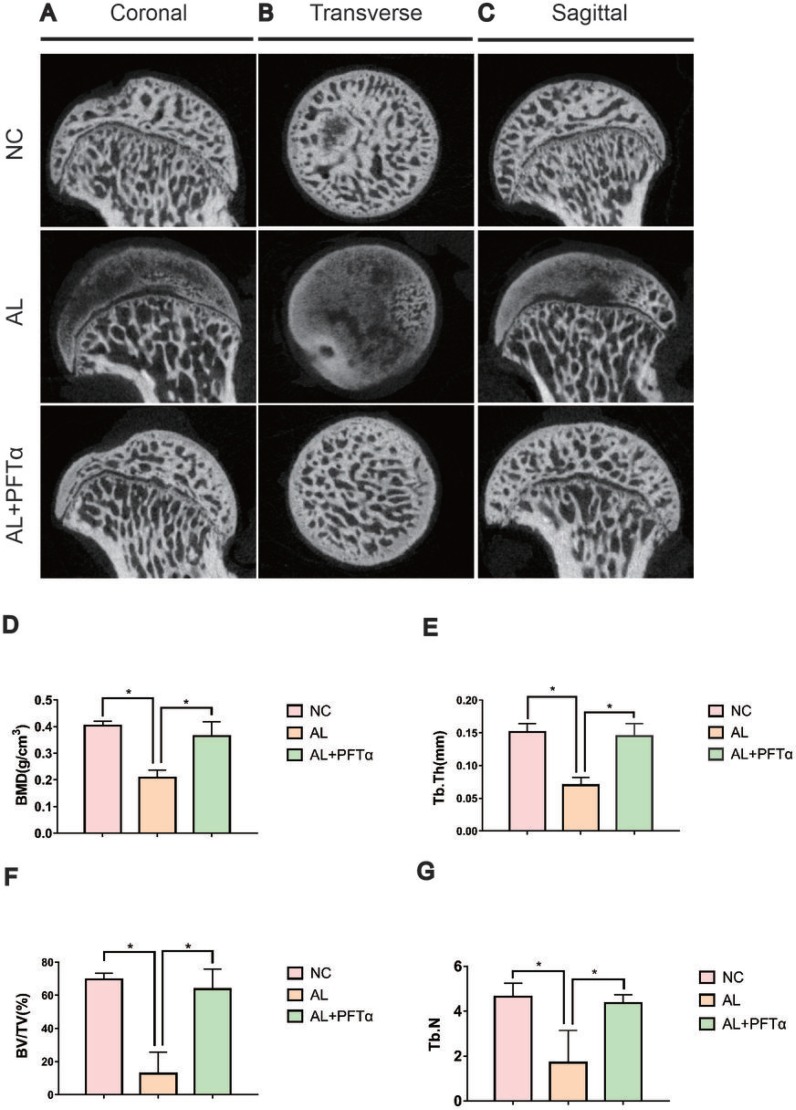
Alcohol-induced ONFH was alleviated with PFTα treatment by Micro-CT analyses (**A, B, C**) Micro-CT scanning images of the femoral heads were grouped by different sections. Micro-CT images showed significantly less subchondral trabeculae in the AL group compared with that in the NC group, whereas more trabeculae were observed in the AL+PFTα group. (**D, E, F, G**) Values were calculated as the means ± SD. (*N* = 5 for each group; ^*^, significant difference versus the AL group, ^*^p < 0.05) BV/TV: bone volume/tissue volume; Tb.Th: trabecular thickness; Tb.N: trabecular number.

Micro-CT scanning was performed on all sixty femoral heads to quantitatively and qualitatively evaluate the bone tissues within the femoral heads after 6 weeks of the respective treatments. In total, 16 of 20 rats in the AL group showed visible signs of ONFH, whereas only 4 out of 20 rats in the AL+PFTα group had mild ONFH (Figure 7A–7C, Table 1). No rats developed osteonecrosis in the NC group. The micro-CT scanning images supported the results of alcohol-induced ONFH in the rata. Quantitative analyses of the micro-CT parameters further confirmed the effect of alcohol and the efficacy of PFTα in preventing alcohol-induced ONFH in the rats. As shown in Figures 7D-7G, parameters including BMD, BV/TV ratio, Tb.Th and Tb.N in the AL group were significantly reduced compared to those in the NC group (^*^p < 0.05),while supplementation with PFTα significantly increased the BV/TV ratio, Tb.Th and Tb.N of the femoral head compared to that in the AL group (^*^p < 0.05).

**Table 1:** The incidence of alcohol-induced ONFH

	NC	AL	AL+PFTα
ONFH incidence	None	16/20^*,#^	3/20

The incidence of osteonecrosis of femoral heads in each group (*N* = 20 for each group. ^*^significant difference AL vs. NC group, *p* < 0.05; #significant difference AL vs. AL+ PFTα group, *p* < 0.05)

### Fluorochrome labeling analysis demonstrated the beneficial effects of PFTα against alcohol-induced ONFH in rats

**Figure 8:**
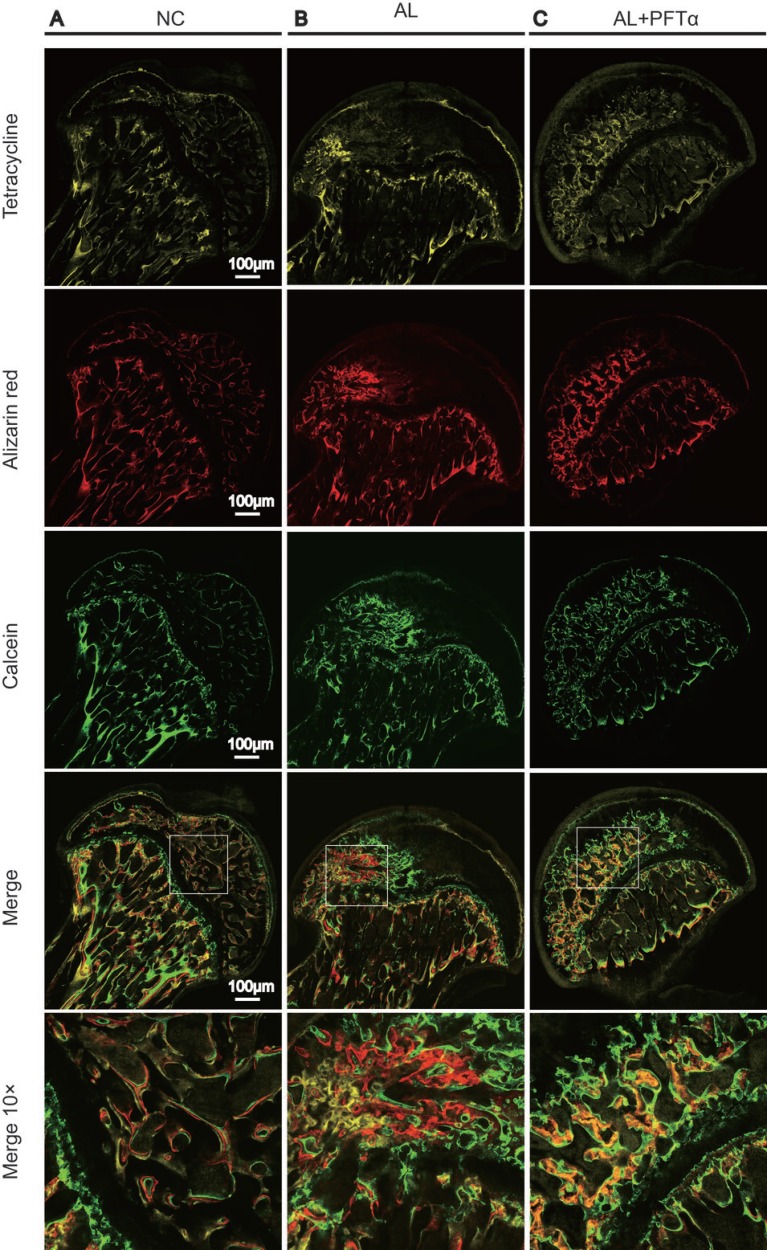
The blockage effect of PFTα against alcohol-induced anti-osteogenesis rat femoral head The fluorochrome labeling analysis showed significantly decreased bone formation in the femoral heads and the process of self-repair. (**A**) Tetracycline (yellow, 2 weeks), alizarin red (red, 4 weeks) and calcein (green, 6weeks) were all found to be deposited onto a broader area of subchondral femoral head trabecular bone in the NC group. (**B**) Fewer subchondral areas of the femoral head were stained with tetracycline, alizarin red and calcein, suggesting decreased new bone formation in the femoral head. (**C**) PFTα injection reversed the inhibitory effect of ethanol on new bone formation in rat femoral heads. Tetracycline, alizarin red and calcein were deposited onto larger areas of subchondral trabecular bone than what was observed in the AL group.

To examine bone formation and mineralization in the femoral heads at different time points, we performed fluorochrome labeling with tetracycline, Alizarin Red and calcein. The results indicated that in the control group, tetracycline (yellow, 0 to 2 weeks), Alizarin Red (red, 2 to 4 weeks) and calcein (green, 4 to 6 weeks) were all deposited onto a broader area of the subchondral femoral head trabeculae (Figure 8A). However, with alcohol administration, we found less subchondral femoral head area staining with tetracycline, alizarin red and calcein, indicating decreased bone formation and increased bone resorption in the femoral head compared to that in the NC group (Figure 8B). Interestingly, a large area of subchondral trabecular bone stained with calcein (green, 6 weeks) and a small area stained with Alizarin Red (red, 4 weeks) and tetracycline (yellow, 2 weeks) showed the least amount of area. This observation might indicate the process of self-repair of the femoral head subchondral trabecular bone, which was observed by other investigators [33] (Figure 8B). With PFTα injection, we found increased tetracycline, alizarin red and calcein deposited onto a broader area of subchondral trabecular bone than what was observed in the AL group, indicating less bone resorption and increased bone formation of the femoral head tissue (Figure 8C).

## DISCUSSION

Alcohol is known to be one of the leading contributors to ONFH [9, 41, 42], along with other risk factors, including steroid use, lipid metabolism abnormalities, thrombosis, and oxidative stress [43]. However, the precise pathological mechanism of alcohol-induced ONFH remains unclear. Previous investigators established an animal model of alcohol-induced femoral head osteonecrosis using the alcohol-containing Lieber-DeCarli diet [44]. We therefore based our *in vivo* study on this animal model.

In human studies, ONFH was often accompanied by a significant decrease in osteogenesis [45–47]. Previous studies demonstrated that ethanol impaired the osteogenic differentiation of BMSCs via the suppression of the Wnt/β-catenin pathway [48–51]. Additionally, the p53 gene was known to exert a negative effect on the osteogenic differentiation of BMSCs [4, 21]. Previously, massive crosstalk between p53 and the Wnt/β-catenin pathway was observed by other researchers [52–57]. Based on these findings, we examined the associations among p53, osteogenesis, ethanol, and Wnt/β-catenin.

Briefly, in an *in vitro* study, we found that the ethanol-induced anti-osteogenic effect was associated with suppression of the Wnt/β-catenin pathway via activated p53 in hBMSCs, which was attenuated by PFTα. This result was consistent with previous studies [56]. Furthermore, we found that ethanol-induced GSK3β deactivation was also restored by PFTα, which contributed to the accumulation of β-catenin in the cytoplasm and subsequent translocation to the nucleus. A similar finding was also observed in previous studies [14]. The use of the selective p53 activators Tenovin-1 and NSC207895 could increase the protein expression of p53 as well as its phosphorylation, indicating activation of the p53 pathway [22]. For downstream effectors, the decreased phosphorylation of GSK3β and β-catenin indicated that p53 activation partially mimicked the effect of ethanol on the p53/Wnt/β-catenin pathway in hBMSCs. In a previous study, PFTα was shown to decrease the stability of p53, which could be more easily degraded, and therefore decrease p53 activity [58]. Li *et al.* also reported that PFTα blocked p53 accumulation and activation, resulting in decreased p53-induced apoptosis [59]. In the current study, we found that both p53 and p-p53 were decreased by PFTα, probably due to increased p53 degradation and decreased phosphorylation [60]. PFTα also plays an important role in the regulation of p53-mediated transcriptional activation [61], which might be another mechanism in the protective effect of PFTα against ethanol. Further study and exploration is needed in future. Thus, we concluded that PFTα attenuated ethanol-induced p53 activation and GSK3β dephosphorylation, which restored β-catenin levels in both the cell nucleus and cytoplasm and promoted the transcription of osteogenic-related proteins.

In *in vivo* experiments, PFTα was co-administered with alcohol in rats. ONFH was defined as diffuse empty lacunae or the presence of pyknotic nuclei in bone trabeculae accompanied by surrounding bone marrow cell necrosis as previously reported [34–36]. A significantly lower ONFH incidence was observed in the AL+PFTα group than in the rats that received ethanol alone, as indicated by the results of H&E staining and micro-CT analysis. As previously reported, apoptosis plays an important role in the development of ONFH [62]. Our cleaved caspase-3 immunostaining and TUNEL results provided evidence of osteonecrosis in the femoral heads and the alleviation of this condition by PFTα. Additionally, using immunofluorescence staining, higher expression of osteogenic-related proteins, including OCN, OPN, RUNX2 and COL1, in the femoral head subchondral bone was observed in the rats that were co-administered PFTα than in the rats fed alcohol alone, indicating higher osteogenic activity induced by PFTα. Notable findings were observed using tetracycline, alizarin red and calcein in sequence to label new bone formation. We found that the tetracycline-labeled (yellow, 2 weeks) subchondral trabeculae were significantly destroyed compared to the Alizarin Red-labeled (red, 4 weeks) and calcein-labeled (green, 6 weeks) subchondral bone in the AL group. This sequence might contribute to the remodeling characteristics of bone [63–65]. The decreased bone formation in the early stage of ONFH (2 weeks) and the increased new bone formation in the late stage of ONFH (6 weeks) showed the self-repair and reconstruction process of subchondral trabecular bone. Similar results were observed by other investigators [66–68]. However, rats that were co-administered PFTα showed significantly less resorption of tetracycline or Alizarin Red-labeled subchondral trabeculae, indicating its anti-ethanol effect on osteogenesis (Figure 8).

Furthermore, PFTα appeared to be a safe drug. Previously, PFTα was reported to be beneficial for its protection against side effects in cancer therapy [69], the regenerative repair of cerebral ischemia [70, 71], Parkinson’s disease and Alzheimer’s disease [72] and hepatocyte necrosis [73]. In this study, no significant changes in the body weight, appearance, and behavior of the rats were observed after PFTα treatment (1 mg/kg/d). The liver, brain, lungs and kidneys of the PFTα-treated rats were examined, and no signs of tumorigenesis were observed (Supplementary Figure 1).

In conclusion, we believe that the ethanol-induced anti-osteogenic effect was associated with the p53-related activation of β-catenin degradation and the inhibition of the Wnt/β-catenin pathway. This action of ethanol contributes to the development of alcohol-induced ONFH, and the deactivation of p53 by PFTα treatment might be an important therapeutic strategy to prevent alcohol-induced ONFH.

## MATERIALS AND METHODS

### Cell culture

Human bone mesenchymal stem cells (hBMSCs) were harvested from the femoral heads of donors who underwent hip arthroplasty surgery according to the method described previously [74]. The hBMSCs were cultured using α-MEM (Gibco BRL, Grand Island, NY, USA) supplemented with 10% FBS (Invitrogen, Carlsbad, CA, USA), 1% penicillin and streptomycin (Gibco, Carlsbad, CA, USA) and maintained at 37°C with 5% CO_2_. The passage of hBMSCs was performed when they reached a confluence of 70–80%. The hBMSCs of five to seven passages were used in all experiments. This study was approved by the Institutional Ethics Review Committee at Shanghai Sixth People’s Hospital. Informed consent was obtained from all subjects. All experiments on hBMSCs were performed according to approved guidelines and regulations.

### Cell toxicity and proliferation

The effect of ethanol and PFTα on the proliferation of BMSCs was assessed using a cell viability assay (Cell Counting Kit-8 (CCK-8); Dojindo Molecular Technologies, Inc., Japan). Briefly, BMSCs were seeded in 96-well plates at an initial density of 5 × 10^3^ cells/well and cultured in 50 mM of ethanol and/or different concentrations of PFTα (0.1 μM, 1 μM and 10 μM) for 1, 3, 5 and 7 days. Then, 20 μl of CCK-8 solution and 180 μl of culture medium were added to each well at each time point and incubated for 2 hours at 37°C. Aliquots (100 μl) were then obtained from each well and transferred to another 96-well plate. The absorbance of the samples was measured at 450 nm using a spectrophotometric microplate reader (Bio-Rad 680, USA). The results were expressed as the optical density of the aliquots minus the absorbance of the blank wells.

### ALP activity

To measure ALP activity, 1 × 10^5^ cells per well were seeded on 48-well plates and grown to 90% confluence within 48 hours. Next, the culture medium was replaced with α-MEM supplemented with 10% FBS, 1% penicillin and streptomycin, 1 mM of ascorbyl-2-phosphate, 4mM L-glutamine and 100 U/mL each of penicillin and streptomycin (Gibco, Carlsbad, CA), which is the condition known to drive osteogenic differentiation. The osteogenic differentiation medium was refreshed daily. The ALP activity was measured at multiple time points (7 and 14 days) using a microplate test kit (Nanjing Jiancheng Bioengineering Institute, Nanjing, China).

### RT-PCR

The gene expressions of RUNX2, OCN, COL-1 were measured using qRT-PCR. RNA was extracted from the cells using Trizol reagent (Invitrogen). Then, RNA was reverse-transcribed into complementary DNA (cDNA) using EasyScript one-step gDNA Removal and cDNA Synthesis Supermix (TransGen Biotech, Beijing, China). The qRT-PCR analysis was performed on the ABI HT7900 (Applied Biosystems, Australia) using the TransStart Tip Green qPCR SuperMix (TransGen Biotech) according to the manufacturer’s instructions. The relative expression of the genes was normalized to β-actin. The forward and reverse primers (BioTNT, Shanghai, China) are listed as follows: OCN: 5′ AGC CTT TGT GTC CAA GCA 3′ 5′ CCA GCC ATT GAT ACA GGT AG 3′; Runx2: 5′ TAA TCT CCG CAG GTC ACT AC 5′ CTG AAG AGG CTG TTT GAT G; and Collagen I: 5′ GAC ATC CCA CCA ATC ACC TG 5′ CGT CAT CGC ACA ACA CCT T.

### Western blotting

The proteins were obtained from the hBMSCs using a cell lysis buffer supplemented with a proteinase inhibitor. The amount of protein was measured using the BCA protein assay kit (Cell Signaling Technology, Danvers, MA). A total of 20 μg of protein were resolved on SDS-PAGE gels and then transferred to PVDF membranes. The protein was blocked with 5% milk in Tris-buffered saline 0.1% Tween (TBST). The membranes were incubated with primary antibodies. Primary antibodies including anti-β-catenin, anti-Runx2, anti-p-GSK3β, and anti-total-GSK3β were provided by Cell Signaling Technology, Shanghai, China. The membranes were then incubated with an anti-rabbit and anti-mouse secondary antibody (Cell Signaling Technology). After chemiluminescence, the LEICA DM 4000 was used to detect the target bands. The protein levels were normalized by β-actin (Cell Signaling Technology).

### Preparation of the nucleus and cytosolic extracts

The NE-PER Nuclear and Cytoplasmic Extraction Reagents Kit (Pierce Biotechnology, Rockford, IL, USA) was used for the stepwise separation of the protein extracts from the cell nucleus and cytoplasm. The protein concentrations were determined using a BCA protein assay kit (Cell Signaling Technology, Shanghai, China). β-tubulin (CST, Shanghai, China) was used as an internal reference for the cytoplasmic proteins, and Histon-3 (CST, Shanghai, China) was used as the normalization control for the nuclear proteins.

### Immunofluorescent staining

Cells were seeded on 0.1% gelatin-coated glass cover slips that were placed on a 6-well plate and allowed 48 hours for adherence. Then, the cells were incubated in culture medium supplemented with ethanol (50mM) and/or different concentrations of PFTα as indicated. The cells were fixed using 4% (wt/vol) paraformaldehyde for 20 min, permeabilized with 0.3% Triton X-100 in PBS for 15 min, and incubated with anti-OCN (Proteintech, Shanghai, China), anti-OPN (Proteintech, Shanghai, China), and anti-COL1 (Proteintech, Shanghai, China) at 4°C overnight. After being washed with PBS three times, the cells were incubated for 1 hour with the Alexa fluor^™^488 secondary antibody (Invitrogen) and 4’, 6-diamidino-2-phenylindole (DAPI) for nuclear staining. The cells were then rinsed with PBS and the immunofluorescence images were captured using an immunofluorescence microscope.

### Animal grouping and treatment

Sixty 8-week-old male Sprague-Dawley rats were used in this study. All procedures were performed with the approval of the Animal Research Committee at Shanghai Sixth People’s Hospital. The account number is 2017–0034. Rats were randomly and evenly divided into three groups as follows: (1) normal control group (NC), (2) alcohol group (AL), and (3) alcohol + PFTα group (AL+PFTα). For 6 weeks, the rats in the AL group received an ethanol-containing Lieber-DeCarli liquid diet (FBSH, Shanghai, China), which contained 8% (weight/volume) ethanol (36% of daily calories), fat (38% of daily calories) and protein (17% of daily calories). The rats in the AL+PFTα group received the same ethanol-containing diet for 6 weeks and were co-treated with PFTα (1mg/kg/d) by intraperitoneal (i.p) injection. The control rats were fed the Lieber-DeCarli diet without ethanol (maltodextrin was substituted for the ethanol, which contains the same amount of calories) for 6 weeks. All rats were fed the Lieber-DeCarli diet one week prior to the start of the experiment for adaptation. Dietary intake was strictly limited to the lowest amount consumed by any group one day before to minimize the difference in results from food intake. Within each group, the rats had free access to a liquid diet throughout the study as was performed previously by Okazaki et al. [33]*.* Diets were prepared fresh daily. To detect new bone formation and mineralization, fluorescence labeling was performed via intraperitoneal injections. Briefly, tetracycline (20 mg/kg, Sigma-Aldrich, China), calcium green (10 mg/kg, Sigma-Aldrich, China) and alizarin red S (30 mg/kg, Sigma-Aldrich, China) were injected at the beginning of the experiment and during the second week and fourth week of the experiment. All rats were sacrificed under anesthesia at the end of the 6th week. The left femurs were immediately excised and fixed in 4% paraformaldehyde for 72 hours.

### Micro-CT scanning

Micro-CT scanning of all sixty femoral heads was performed using a micro-CT scanner set at a 9-micron voxel size. Image acquisition was performed at 35 kVof energy and 220mA of intensity. Image reconstruction was performed using CTVol software. The bone mineral density (BMD), trabecular bone volume fraction (BV/TV), trabecular thickness (Tb.Th) and trabecular number (Tb.N) were then calculated.

### Histological staining

Decalcification of several specimens was performed using EDTA with constant shaking for one month. After decalcification, the bone tissue was embedded in paraffin and cut into 5-mm-thick sections. To evaluate the subchondral trabecular structure, five specimens were stained with hematoxylin & eosin (H&E) and examined using the LEICA DM 4000 microscope to assess the overall morphology of the femoral heads. Three specimens were deparaffinized, and the antigens were retrieved and incubated with anti-caspase-3-cleaved antibody (CST, Shanghai, China).The antigens were then incubated with a secondary antibody and 3, 30-diaminobenzidine (DAB). Images were captured with the LEICA DM 4000 microscope.

### Immunofluorescence staining

Three specimens from each group were deparaffinized, blocked with 1% BSA and incubated with anti-RUNX2 (CST, Shanghai, China), anti-OCN (Proteintech, Shanghai, China), anti-OPN (Proteintech, Shanghai, China) and anti-COL1 (Proteintech, Shanghai, China) primary antibodies for 2 hours at room temperature and then treated with a secondary antibody for 1 hour at room temperature. To visualize the cell nucleus, 4′, 6-diamidino-2-phenylindole (DAPI) was used. Immunofluorescence images were captured using an immunofluorescence microscope and analyzed using the Image-Pro Plus software. The protein integrated option density (IOD) and the total area of trabecular bone were measured, and the mean density (IOD/area) was calculated and counted. For the fluorochrome-labeled assay, freshly dissected femoral heads were fixed in 4% paraformaldehyde for 72 hours. Five specimens were dehydrated in a graded series of alcohol and then embedded in polymethylmethacrylate. The samples were sectioned into 150-μm-thick specimens using a saw microtome. The sections were glued onto a transparent plastic plate. The images of the fluorescent-labeled specimens were captured using a confocal laser scanning microscope (Leica, Heidelberg, Germany). The excitation/emission wavelengths were set as follows: 405/560–590 nm (tetracycline, yellow), 543/580–670 nm (alizarin red, red) and 488/500–550 nm (calcium green, green) [75].

### TUNEL

For TdT-mediated dUTP nick end labeling (TUNEL), three specimens of each group were deparaffinized, and antigens were retrieved. The procedure was performed using a TUNEL staining kit (Roche, Shanghai, China) according to the manufacturer’s instructions. Images were captured using the LEICA DM 4000 microscope.

### Inhibitors and agonists

PFTα, Tenovin-1 and NSC207895 were purchased from Selleck (Shanghai, China). hBMSCs were cultured in a medium containing a concentration of PFTα, Tenovin-1 (10 μM), and NSC207895 (10 μM) for the *in vitro* experiments.

### Statistical analysis

All experiments were done in triplicate. Data were presented as mean ± SE (standard error). Statistical analysis was performed using SPSS 20.0 software. Comparisons between more than two groups were analyzed using one-way analysis of variance (ANOVA), with subsequent Bonferroni correction. *P* values of less than 0.05 were considered statistically significant.

## SUPPLEMENTARY MATERIALS FIGURE


