# Supplementary material for: The protective effect of PFTα on alcohol-induced osteonecrosis of the femoral head
Source: Oncotarget. 2017 Jul 11;8(59):100691–707. doi: 10.18632/oncotarget.19160 (PMC5725055; doi:10.18632/oncotarget.19160)
Supplement: Supplementary file 1 [file oncotarget-08-100691-s001.pdf]

The protective effect of PFTα on alcohol-induced osteonecrosis of the femoral head

SUPPLEMENTARY MATERIALS

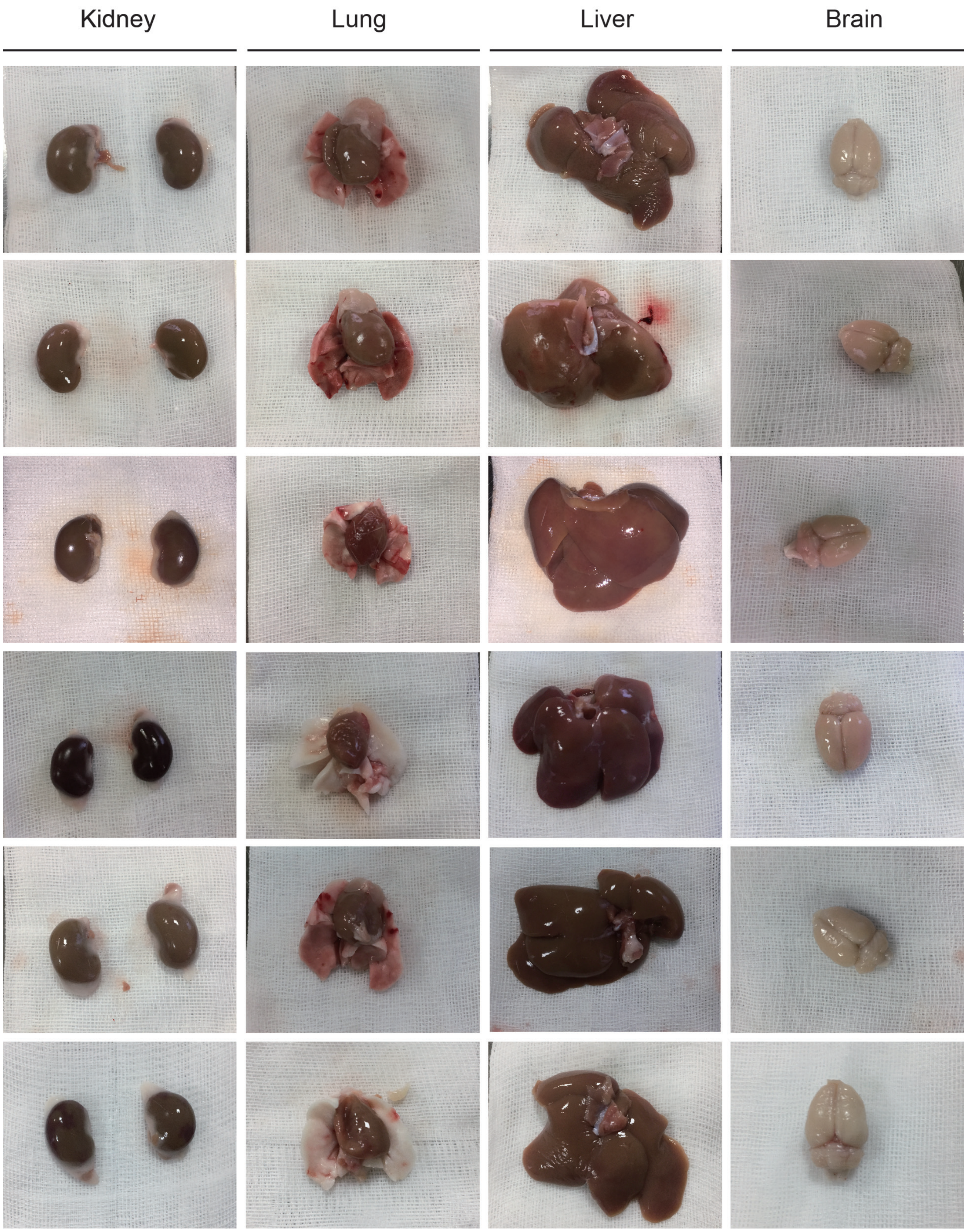

Supplementary Figure 1: General assessment of organs from the PFTα-treated rats. Six out of twenty rats were randomly chosen from PFTα-treated group. The brain, lungs, liver and kidneys were harvested and examined. No sign of tumorigenesis was observed.
